# Gill ectoparasites of *Barbus martorelli* (Teleostean: Cyprinidae) from a tropical watercourse (Cameroon, Africa): conflict or coexistence?

**DOI:** 10.1051/parasite/2011181071

**Published:** 2011-02-15

**Authors:** J. Tombi, C.F. Bilong Bilong, S. Morand

**Affiliations:** 1 Laboratory of Parasitology and Ecology, Faculty of Science, University of Yaoundé I P.O. Box 812 Yaoundé Cameroon; 2 Institut des Sciences de l’Évolution, CNRS – UM2, CC65, Université de Monpellier 2 34095 Montpellier France

**Keywords:** *Barbus martorelli*, gill parasites, coexistence, infracommunity, filament, filament fraction, isolationist, *Barbus martorelli*, parasites branchiaux, infracommunauté, filament, fraction de filament, isolationniste

## Abstract

The structure and stability of parasite communities have been mainly explained by high diversity and strong interactions among parasite species. During 16 months, 558 *Barbus martorelli* gill infracommunities were studied in a tropical zone to determine whether parasite infrapopulations interact. Three levels were retained: the infracommunity level, the gill filament level, and the filament fraction level. Single species infections in *Barbus martorelli* were very rare and only concerned the core species: *Dactylogyrus bopeleti*, *D. insolitus*, *D. simplex* and *Myxobolus barbi.* Mixed infections appeared as a general rule in this fish species. Interspecific interactions at all three levels were statistically non significant. Our results suggest that *Barbus martorelli* gill parasites are non interactive (isolationist).

## Abstract (fr)

La structure et la stabilité des communautés de parasites sont expliquées principalement par la diversité spécifique ou par les interactions entre espèces parasites. Durant 16 mois, les infracommunautés parasitaires branchiales de 559 *Barbus martorelli* ont été étudiées dans une zone intertropicale afin de déterminer si les populations de ces parasites sont en interaction. Trois niveaux d’analyse ont été retenus : l’infra-communauté, le filament et la fraction du filament branchial. Les infections par une seule espèce de parasite sont rares et concernent les espèces dominantes : *Dactylogyrus bopeleti*, *D. insolitus*, *D. simplex* et *Myxobolus barbi.* Les infections multiples sont la règle chez ce poisson. Les interactions interspécifiques ne sont pas statistiquement soutenues, ce qui suggère que les populations de parasites branchiaux de *Barbus martorelli* forment des communautés non interactives ou isolationnistes.

## Introduction

Community ecology of fish parasites has been the subject of numerous studies since the early works of Dogiel and colleagues in the 1940 s (see Dove, 1999, but also Koskivaara *et al*., 1992; Rohde *et al*., 1995; Sasal & Morand, 1998). These studies have mostly concerned temperate fresh water fish (Šimková et al., 2002a, Šimková, Kadlec 2002b) and temperate or tropical marine parasites (Benajiba *et al*., 1994; Rohde *et al*., 1995; Lo *et al*., 1998; Sasal & Morand, 1998; Gonzalez & Poulin, 2005). However, studies concerning freshwater fish in intertropical zone and particularly in Africa are rare (Obiekezie, 1991; Obiekezie & Taege, 1991; Bilong Bilong & Njiné, 1998; Olurin & Somorin, 2006). As far as the parasite community interactions are concerned, some theoretical principles are proposed. The structure of parasite communities has been explained in different and sometimes contradictory ways. For Holmes (1973), the structure of parasite communities is the result of competitive interspecific interactions that act on the population’s spatial distribution and density (interactive communities). In contrast, for Price (1980) the structure of parasite communities may result from the random assembly of species that evolve independently (isolationist communities). Studying fish ectoparasites, Rohde (1991) concluded that the microhabitats of a given parasite species did not seem to be affected by the presence of potential competitors. However, except for a few isolated reports that showed positive interactions (Adams, 1986; Silan & Maillard, 1990; Koskivaara, 1992), most interspecific studies showed the lack of interactions in parasite communities (El Hafidi *et al*., 1998; Morand & Šimková, 2005). Generally, interactive communities are species rich, have many core species (often specialist), a large degree of niche overlap between parasite species, and a dominance of interspecific interactions over individualistic responses, resulting in predictable infracommunities (Holmes & Price, 1986). Isolationist communities generally have few species, a higher proportion of rare (satellite) generalist species, large amounts of available niche space, and dominance of individualistic responses over interspecific interactions. This results in communities which structure is difficult to predict and largely stochastically determined (Dove, 1999). Finally, greater parasite diversity represents more stable communities.

*Barbus martorelli* Roman, 1971 (Cyprinidae) is one of the common fish species in Southern Cameroon (Vivien, 1991). Highly consumed by rural human populations in this part of the nation, it thus constitutes one of main animal proteins source. The gills of this fish are parasitized by two species of Myxosporidia: *Myxobolus barbi* Fomena, Bouix and Birgi, 1985 and *M. njinei* Fomena, Bouix and Birgi, 1989 (Fomena *et al*., 1989) and eight species of Monogenea: *Dactylogyrus bopeleti*, *D. insolitus*, *D. simplex*, *D. maillardi*, *Dogielius martorellii* (Birgi & Lambert, 1987), *Dogielius* sp., *Gyrodactylus* sp. and Polystomatid larva (Tombi & Bilong Bilong, 2004). Aspects of the distribution of all the above gill parasites and the temporal structure of their communities were studied by Tombi & Bilong Bilong (2004) and Bilong Bilong & Tombi (2005) respectively. Taking in consideration this high parasite diversity, the present work aims to study the composition of different gill communities and determine whether or not the different populations are isolationist or interactive.

## Material and Methods

Host specimens were caught from May 1998 to August 1999, in the Foulou watercourse at Nkolfoulou 3°53’N, 11°34’E *i.e.* in the outskirts of Yaoundé, Capital of the Republic of Cameroon (Central Africa). Fish standard lengths (SL) were measured from the extremity of the muzzle to the last vertebra. Methods of catching, transport, dissection, parasite collection have been described by Tombi & Bilong Bilong (2004). Three levels of analyses, from the large to the most reduced, were retained in the present study of parasite associations, *i.e.* the infracommunity, the gill filament and the filament fraction levels respectively. The latter was obtained by subdividing a filament in three equidistant parts (proximal, median, and distal fractions), using an ocular micrometer of the stereoscopic microscope. The terms infrapopulation and infracommunity on the one hand, compound and component community on the other hand, are defined according to Sousa (1994) and Combes (1995). The prevalence and intensity of parasites were defined according to Margolis *et al*. (1982). On the prevalence basis and according to Valtonen *et al*. (1997), parasite species were termed common (occurrence > 50%), intermediate (10% ≤ occurrence ≤ 50%) and rare (occurrence < 10%). These categories correspond to what Hansky in Koskivaara & Valtonen (1991) designated as core, secondary or satellite. It should be noted that rare species were hereafter excluded from the inter-action analysis due to their rare occurrence and very low intensities: less than two. Furthermore and according to Rohde (1979), Holmes, (1987), and Combes, (1995), rare species are not structuring.

### Quantifying community structure using null models

Dice’s coefficient (D) and that of Whittaker & Fairbanks (WF) were used to measure the degree of association. Dice coefficient is a similarity measure related to the Jaccard index. The percentage similarity index of Whittaker & Fairbanks incorporates information about all of the species that are counted and, theoretically, values for this index will range from 0.0 (totally different communities) to 100 (identical communities). Forbes’ index measured the amount of association deviation from expectation (Abdallah & Saporta, 2003). The Chi-square (χ^2^) test was used to evaluate the degree of departure from random association and P values less than 5% were considered significant. The abbreviation “d.f.” was used for degree of freedom. We tested whether co-occurrence of different monogenean species in the same fish host were more or less frequent than expected by chance, we followed Janovy *et al*. (1995) by generating a null model of expected frequencies based on the actual prevalence of the different parasite species. The null model corresponds to the probability that any two or more parasite species co-occurring in a fish is equal to the product of their prevalence in the sample. Observed and expected frequencies were compared with a Chi-square (χ^2^) test (Janovy *et al*., 1995).

## Results

A total of 558 *Barbus martorelli* were caught and dissected from May 1998 to August 1999. Their standard length (SL) varied from 30 to 119 mm. The prevalences of helminth species were 80.6%, 84.4%, 70.8%, 33.2%, 9.3%, 0.4%, 5.9%, and 5.6% for *D. bopeleti*, *D. insolitus*, *D. simplex*, *D. maillardi*, *Dogielius* sp., *Do. martorelli*, Polystomatid larva and *Gyrodactylus* sp. respectively; the occurrence of myxosporidian cysts was 79% and 27.7% for *M. barbi* and *M. njinei* respectively. Thus, *D. bopeleti*, *D. insolitus* and *D. simplex* represented the helminth core species, while *D. maillardi* was a secondary one and *Dogielius* sp., *Do. martorelli*, Polystomatid larva and *Gyrodactylus* sp. revealed rare. Diffuse spores being uncountable, based on the occurrence of cysts, *M. barbi* and *M. njinei* were considered core and secondary metazoan species respectively.

### Infracommunity

Four (0.7%) of 558 *B. martorelli* were not parasitized while 554 (99.3%) were infected by at least one parasite species. A total of 8,773 helmiths and 44,824 cysts were collected with an average of 16.56 ± 15.02 and 98.79 ± 203.58 parasites/fish respectively. 24 host individuals (4.3%) showed a single species infection and 42 (7.6%), 119 (21.5%), 179 (32.3%), 131 (23.7%), 50 (9.0%) and 9 (1.6%) had multiple infections with 2, 3, 4, 5, 6 and 7 parasite species respectively (Table I). *Myxobolus njinei* and none of the rare species were found solely. A total of 95 of the 1,023 expected combinations were observed (Table I). *Myxobolus barbi*, alone, parasitized 18 host individuals. The following combinations “*D. insolitus – M. barbi*”, “*D. bopeleti – D. insolitus – M. barbi*”, “*D. bopeleti – D. insolitus – D. simplex – M. barbi*”, “*D. bopeleti – D. insolitus – D. maillardi – D. simplex – M. barbi*”, “*D. bopeleti – D. insolitus – D. maillardi – D. simplex – M. barbi – M. njinei*” and “*D. bopeleti – D. insolitus – D. maillardi – D. simplex – Do. martorelli – M. barbi – M. njinei*” were the most frequent for multiple infections by 2, 3, 4, 5, 6 and 7 different species respectively (Table I). In Nkolfoulou, two parasite combinations: “*D. bopeleti – D. insolitus – D. simplex – M. barbi*” and “*D. bopeleti – D. insolitus – D. maillardi – D. simplex – M. barbi*” amongst 95 observed had intermediate occurrence # 15.2% and # 10.3% respectively; the other remaining assemblages were rare. Also, none of the 544 *B. martorelli* harboured eight to ten parasite species.

**Table I.** Types of combinations among gill parasites.

		Type of combination	Number of hosts	Host percentage
		***D. bopeleti***	2	0.36
		***D. insolitus***	1	0.18
Single infection		*D. maillardi*	1	0.18
		***D. simplex***	2	0.36
		***M. barbi***	**18**	**3.25**

	By 2 parasite species	*D. bopeleti — D. insolitus*	6	1.08
		*D. bopeleti — D. simplex*	1	0.18
		*D. bopeleti — M. barbi*	4	0.72
		*D. insolitus — D. maillardi*	2	0.36
		*D. insolitus — D. simplex*	7	1.26
		*D. insolitus — Dogielius* sp.*	1	0.18
		***D. insolitus — M. barbi***	**9**	**1.62**
		*D. maillardi — D. simplex*	1	0.18
		*D. simplex — M. barbi*	8	1.44
		*D. simplex — Gyrodactylus* sp.*	1	0.18
		*Gyrodactylus* sp.* *— M. barbi*	1	0.18
		*Do. martorelli* * *— M. barbi*	1	0.18
	
	By 3 parasite species	*D. bopeleti — D. insolitus — D. maillardi*	3	0.54
		*D. bopeleti — D. insolitus — D. simplex*	25	4.51
		***D. bopeleti — D. insolitus — M. barbi***	**36**	**6.50**
		*D. bopeleti — D. insolitus — M. njinei*	2	0.36
		*D. bopeleti — D maillardi — D. simplex*	2	0.36
Multiple infections		*D. bopeleti — D. simplex — M. barbi*	10	1.81
		*D. bopeleti — M. barbi — M. njinei*	6	1.08
		D. bopeleti — D. insolitus — Do. martorelli *	1	0.18
		*D. bopeleti — Do. martorelli* * *— M. njinei*	1	0.18
		*D. insolitus — D. maillardi — D. simplex*	2	0.36
		*D. insolitus — D. maillardi — M. njinei*	1	0.18
		*D. insolitus — D. simplex — M. barbi*	17	3.07
		*D. insolitus — D. simplex — M. njinei*	2	0.36
		*D. insolitus — M. barbi — M. njinei*	4	0.72
		*D. insolitus — Do. martorelli* * *— Gyrodactylus* sp.*	1	0.18
		*D. insolitus — Do. martorelli* * *— M. barbi*	1	0.18
		*D. insolitus — Dogielius* sp.* *— M. barbi*	1	0.18
		*D. maillardi — D. simplex — M. barbi*	2	0.36
		*D. simplex — M. barbi — M. njinei*	2	0.36
	
	By 4 parasite species	*D. bopeleti — D. insolitus — D. maillardi — D. simplex*	24	4.33
		*D. bopeleti — D. insolitus — D. maillardi — M. barbi*	6	1.08
		*D. bopeleti — D. insolitus — D. maillardi — M. njinei*	1	0.18
		***D. bopeleti — D. insolitus — D. simplex — M. barbi***	**84**	**15.16**
		*D. bopeleti — D. insolitus — D. simplex — M. njinei*	3	0.54
		*D. bopeleti — D. insolitus — M. barbi — M. njinei*	19	3.42
		*D. bopeleti — D. maillardi — D. simplex — M. barbi*	4	0.72
		*D. bopeleti — D. maillardi — M. barbi — M. njinei*	1	0.18
		*D. bopeleti — D. simplex — M. barbi — M. njinei*	6	1.08
		D. bopeleti — D. insolitus — D. maillardi — Polystomatid*	1	0,18
		D. bopeleti — D. insolitus — D. simplex — Do. martorelli *	2	0.36
		*D. bopeleti — D. insolitus — D. simplex — Dogielius* sp.*	2	0.36
		*D. bopeleti — D. insolitus — Do. martorelli* * *— M. barbi*	1	0.18
		*D. bopeleti — D. insolitus — Dogielius* sp.* *— M. barbi*	2	0.36
		*D. bopeleti — D. insolitus — Gyrodactylus* sp.* *— M. barbi*	4	0.72
		D. bopeleti — D. insolitus — Polystomatid* — M. barbi	1	0.18
		*D. bopeleti — D. simplex — Do. martorelli* * *— M. barbi*	1	0.18
		*D. insolitus — D. maillardi — D. simplex — M. barbi*	5	0.90
		*D. insolitus — D. maillardi — Gyrodactylus* sp.* *— M. barbi*	1	0.18
		*D. insolitus — D. simplex — M. barbi — M. njinei*	7	1.26
		*D. insolitus — D. simplex — Dogielius* sp.* *— M. barbi*	2	0.36
		*D. insolitus — Do. martorelli — M. barbi — M. njinei*	1	0.18
		*D. maillardi — D. simplex — Dogielius* sp.* *— M. njinei*	1	0.18
	
	By 5 parasite species	***D. bopeleti — D. insolitus — D. maillardi — D. simplex — M. barbi***	**57**	**10.29**
		*D. bopeleti— D. insolitus — D. maillardi — D. simplex — M. njinei*	3	0.54
		*D. bopeleti— D. insolitus — D. maillardi — M. barbi — M. njinei*	5	0.90
		*D. bopeleti— D. insolitus — D. simplex — M. barbi — M. njinei*	40	7.22
		*D. bopeleti— D. maillardi — D. simplex — M. barbi — M. njinei*	4	0.72
		*D. bopeleti— D. insolitus — D. maillardi — D. simplex — Dogielius* sp.*	2	0.36
		*D. bopeleti— D. insolitus — D. maillardi — D. simplex — Gyrodactylus* sp.*	3	0.54
		*D. bopeleti— D. insolitus — D. maillardi — Do. martorelli* * *— M. barbi*	1	0.18
		*D. bopeleti— D. insolitus — D. simplex — Do. martorelli* * — Polystomatid*	1	0.18
Multiple infections		*D. bopeleti— D. insolitus — D. simplex — Dogielius* sp.* *— Gyrodactylus* sp.*	2	0.36
		*D. bopeleti— D. insolitus — D. simplex — Do. martorelli* * *— M. barbi*	2	0.36
		*D. bopeleti— D. insolitus — D. simplex — Dogielius* sp.* *— M. barbi*	2	0.36
		*D. bopeleti— D. insolitus — D. simplex — Gyrodactylus* sp.* *— M. barbi*	1	0.18
		*D. bopeleti— D. insolitus — Do. martorelli* * — Polystomatid* — M.barbi	1	0.18
		*D. bopeleti — D. insolitus — Gyrodactylus* sp.* *— M. barbi — M. njinei*	1	0.18
		D. bopeleti— D. insolitus — Polystomatid* — M. barbi — M. njinei	3	0.54
		*D. insolitus — D. maillardi — D. simplex — M. barbi — M. njinei*	3	0.54
	
	By 6 parasite species	***D. bopeleti — D. insolitus — D. maillardi — D. simplex — M. barbi — M. njinei***	**18**	**3.24**
		*D. bopeleti— D. insolitus — D. maillardi — D. simplex — Do. martorelli* * *— M. barbi*	5	0.90
		*D. bopeleti— D. insolitus — D. maillardi — D. simplex — Dogielius* sp.* *— M. barbi*	2	0.36
		*D. bopeleti— D. insolitus — D. maillardi — D. simplex — Gyrodactylus* sp.* *— M. barbi*	4	0.72
		D. bopeleti— D. insolitus — D. maillardi — D. simplex — Polystomatid* — M. barbi	8	1.44
		D. bopeleti— D. insolitus — D. maillardi — D. simplex — Polystomatid* — Gyrodactylus sp.*	2	0.36
		*D. bopeleti— D. insolitus — D. maillardi — Gyrodactylus* sp.* — Polystomatid* — M. barbi	1	0.18
		*D. bopeleti— D. insolitus — D. maillardi — Gyrodactylus* sp.* *— M. barbi — M. njinei*	1	0.18
		*D. bopeleti— D. insolitus — D. simplex — Do. martorelli* * — Polystomatid* — M. barbi	1	0.18
		*D. bopeleti— D. insolitus — D. simplex — Do. martorelli* * *— M. barbi — M. njinei*	5	0.90
		*D. bopeleti— D. insolitus — D. simplex — Dogielius* sp.* — Polystomatid* — M.njinei	1	0.18
		*D. bopeleti— D. insolitus — D. simplex — Dogielius* sp.* *— M. barbi — M.njinei*	1	0.18
		*D. bopeleti— D. insolitus — D. simplex — Gyrodactylus* sp.* — Polystomatid* — M. barbi	1	0.18
	
	By 7 parasite species	*D. bopeleti — D. insolitus — D. maillardi — D. simplex — Do. martorelli* * *— M. barbi — M. njinei*	**3**	**0.54**
		*D. bopeleti— D. insolitus — D. maillardi — D. simplex — Dogielius* sp.* — Polystomatid* — M. barbi	2	0.36
		*D. bopeleti— D. insolitus — D. maillardi — D. simplex — Dogielius* sp.* *— M. barbi — M. njinei*	1	0.18
		*D. bopeleti— D. insolitus — D. maillardi — D. simplex — Gyrodactylus* sp.* *— M. barbi — M. njinei*	1	0.18
		*D. bopeleti— D. insolitus — D. maillardi — Gyrodactylus* sp.* — Polystomatid* — M. barbi — M. njinei	1	0.18
		*D. bopeleti— D. insolitus — D. simplex — Dogielius* sp.* *— Gyrodactylus* sp.* — Polystomatid* — M. barbi	1	0.18

Total			554	100

*D*.: *Dactylogyrus*; *Do*.: *Dogielius*; *M*.: *Myxobolus*; * rare species; in bold: core species and most frequent combination.

### Interspecific interactions

**Fig 1.**
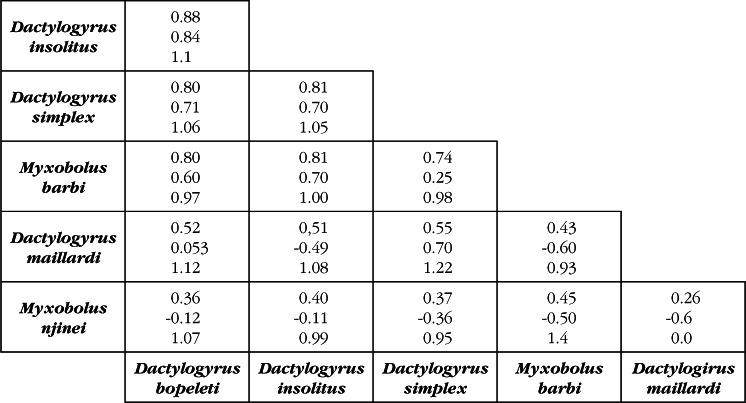
Association coefficients calculated for pairs of parasite species at the infracommunity level. In each box, the first two numbers up to down represent the values of Dice and Whittaker & Fairbanks indexes respectively, and the third number the value of Forbes index.

**Fig 2.**
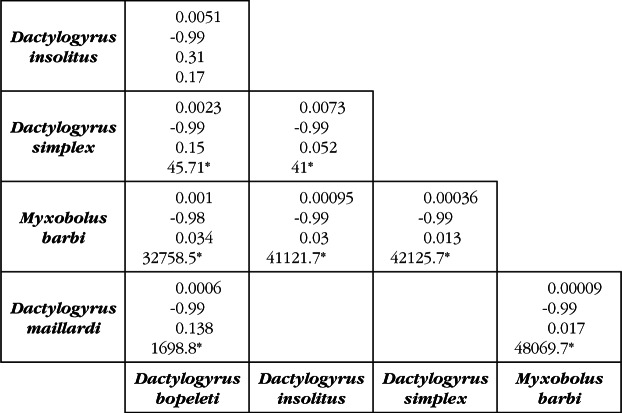
Association coefficients calculated for pairs of parasites species at the filament level. In each box, the first two numbers up to down represent the values of Dice and Whittaker & Fairbanks indexes respectively, while the two latter represent Forbes index and χ^2^ test respectively; * p < 0.05.

At the infracommunity level, all parasite core species showed strong positive associations as D ≥ 0.60 (Fig. 1). The same result was obtained with WF index, except for the pair “*D. simplex – M. barbi*” for which the association was weak (WF = 0.25). Core species and secondary ones never developed strong associations. In all associations observed, the values of F index were almost equal to 1 and due at random, except for *D. maillardi* and *M. njinei* (Fig. 1). Conversely, at the level of a filament (Fig. 2), D varied from 0.9.10^−4^ to 73.10^−4^, supporting obviously that no interaction exist between the different taxa. Forbes’ index varied from 0.013 to 0.31 indicating that, these associations differ from the expected assemblages (P < 0.001), except for the pair “*D. bopeleti – D. insolitus*” (P = 0.88). At the filament fraction level and for the different parasite assemblages, D and WF varied from 0.0065 to 0.013 and -0.99 to -0.95 respectively, proving almost the whole absence of interaction between these parasites.

### Community structure

**Fig 3.**
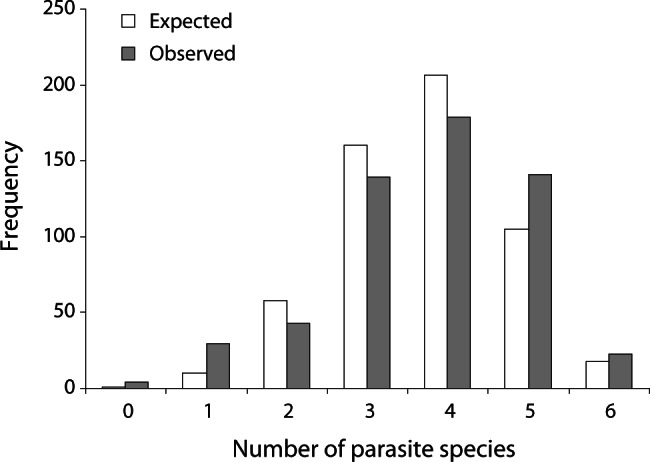
Observed (grey bars) and expected (white bars) frequency distribution of gill parasite species richness at the infracommunity level.

We compared the observed species richness to that predicted by the null model for interactions of parasite species in an assemblage (Janovy *et al*., 1995). We found a significant difference (χ^2^ = 77.17, d.f. = 5, P < 0.0001). Fig. 3 shows this frequency distribution. A significantly greater number of fish carried 1, 5 or 6 (rare species are not considered) parasite species whereas fewer than expected carried 2, 3 or 4 parasite species.

## Discussion

The gill parasite fauna of *Barbus martorelli* is very diversified and made up of two species of Myxosporidia and eight of Monogenea, *i.e.* seven Monopisthocotyleans and one Polyopisthocotylean, all core and secondaries species presenting aggregated distribution (Tombi & Bilong Bilong, 2004). Such observed rich parasitofauna, including many phyla, have been regularly reported in fishes (Koskivaara *et al*., 1991; Benajiba *et al*., 1994; Oliva & Luque, 1998; Tavares & Luque, 2004; Gonzales & Poulin, 2005). In Africa, the study of fish parasite diversity had mainly concerned organisms of the same phylum (Birgi & Lambert, 1987; Fomena *et al*., 1989; Bilong Bilong *et al*., 1999; Bilong Bilong *et al*., 2004; Nack et al., 2005; Nack & Bilong Bilong, 2007).

In *B. martorelli*, the fact that *M. njinei* and all rare species were not observed solely (monospecific infection) suggests that, these parasites may be able to infect their host only when the latter is infected by one or more other parasite taxa. In Cameroon, a similar observation was made for *Cichlidogyrus falcifer* and *Onchobdella aframae*, two monogenean gill parasites of *Hemichromis fasciatus* (Cichlidae) in the Ozum II and Melen pounds (Bilong Bilong, 1995). This could be related to the effects of some parasites on the fish immune responses, which may impact co-infections (Šimková *et al*., 2008; Poisot et al., 2009). This observation and the fact that all but one (98.9%) parasite combinations observed included the core species *D. bopeleti*, *D. insolitus*, *D. simplex* and *M. barbi* confirm that negative interactions did not seem to play an important role in these parasite infracommunities. The use of null model (Janovy *et al*., 1995) confirmed also that parasite species are positively associated rather than negatively. Tombi & Bilong Bilong (2004) showed that the average total number of helminth individuals increased with host size, and was associated with a progressive decrease in the number of cysts, suggesting the existence of negative interspecific interactions between parasite phyla. However, we found no negative association between *Myxobolus barbi* (which formed cysts most abundantly in the different infracommunities studied) and any monogenean species. Therefore, the reduction of this myxosporean cyst abundance as a function of the host length could be associated to some changes related to fish age, such as immune defence (West & Roubal, 1998; Morand *et al*., 2002).

Altogether, our results confirm Rohde’s (1994) opinion that positive interactions between fish ectoparasites are more frequent than negative. Contrary to the statements of Holmes & Price (1986) that negative interactions should be frequent, our results also suggest that infrapopulations of monogeneans in *B. martorelli* are isolationist, although infracommunities are speciesrich and have mainly core species. In isolationist parasite communities where interactions are negligible, the co-occurrence of species in hosts is not expected to deviate from that expected by chance if interspecific interactions are the main structuring processes in parasite infracommunities. Lerssutthichawal & Lim (2005) also stated that the diversity in a community is an indication of stability, *i.e.* the greater the diversity the more stable the community. In conclusion, the infracommunities structure of *B. martorelli* seems to be predictable stable, and isolationist. Therefore, it is worthwhile to determine the spatial niche width of these core species, because it remains unexplained that none of the hosts harboured eight to ten parasite species.
